# Preparation and Immobilization Mechanism on a Novel Composite Carrier PDA-CF/PUF to Improve Cells Immobilization and Xylitol Production

**DOI:** 10.3390/foods13121911

**Published:** 2024-06-18

**Authors:** Le Wang, Jianguang Liu, Yan Shen, Yanli Yin, Zifu Ni, Jun Xi, Yuansen Hu, Qipeng Yuan

**Affiliations:** 1 School of Biological Engineering, National Engineering Research Center of Wheat and Corn Further Processing, Henan University of Technology, Zhengzhou 450001, China; 2 College of Food Science and Engineering, Henan University of Technology, Zhengzhou 450001, China; 3 State Key Laboratory of Chemical Resource Engineering, Beijing University of Chemical Technology, Beijing 100029, China

**Keywords:** xylitol, carbon fiber/polyurethane foam, cells immobilization, biocompatibility

## Abstract

The preparation of a novel composite carrier of polydopamine-modified carbon fiber/polyurethane foam (PDA-CF/PUF) was proposed to improve cell immobilization and the fermentation of xylitol, which is an important food sweetener and multifunctional food additive. *Candida tropicalis* was immobilized on the composite carrier by adsorption and covalent binding. The properties and immobilization mechanism of the composite carrier and its effect on immobilized cells were investigated. It showed that the modification of PDA enhanced the loading of CF on the PUF surface and the adhesion of cells on the composite carrier surface. Also, the biocompatibility of carriers to cells was improved. In addition, the introduction of PDA increased the active groups on the surface of the carrier, enhanced the hydrophilicity, promoted the cells immobilization, and increased the xylitol yield. It was also found that expression of the related gene XYL1 in cells was significantly increased after the immobilization of the PDA-CF/PUF composite carrier during the fermentation. The PDA-CF/PUF was an immobilized carrier with the excellent biocompatibility and immobilization performance, which has great development potential in the industrial production of xylitol.

## 1. Introduction

D-Xylitol (2R,3R,4S)-pentane-1,2,3,4,5-pentol, a natural five-carbon sugar alcohol, is among the most valuable microbial products commonly used as a sweetening agent. The application of xylitol varies widely from food, nutraceuticals, and beverage to pharma industries, making it one of the top 12 global bio-products [1,2] with a rapidly growing market share. Xylitol is considered a “Generally Recognized as Safe” (GRAS) additive by the Food and Drug Administration (FDA) and as a low-calorie sweetener by more than 35 countries [1,3].

The immobilization technology is a method that locates free microorganisms in a limited space area through chemical or physical means to increase the concentration of microbial cells, maintain high biological activity, and reuse them. The immobilized system can be reused in continuous cultures because it can obtain higher cell concentration and shorten the time required for fermentation. It is worth noting that cells immobilization may change the expression level of some genes in microorganisms, thus affecting the physiological function of microorganisms. For example, it was found that the immobilization of *Pseudomonas mendocina GL6* on biochar promoted the expression levels of denitrification functional genes and electron transfer genes involved in denitrification, thus improving its nitrate removal efficiency [4]. Bacterial cellulose was used to immobilize *Haematococcus pluvialis* in some research. The results showed that the key genes related to astaxanthin syntheses, such as *PSY*, *LCY*, *BKT*, and *CHY*, were significantly upregulated after immobilization, with a significant increase in astaxanthin production of *Haematococcus pluvialis* [5]. Cells immobilization has shown great potential in the field of biotechnology. The material-mediated cells immobilization has been widely used in the field of biological fermentation.

As one of the common carbon materials, the carbon fiber (CF) has excellent physical and chemical properties and great biocompatibility, so it has a unique position in the field of cells immobilization technology [6]. The surface of CF was smooth, hydrophobic, non-polar, and chemically inert. Importantly, the direct application of CF fine powder to cells immobilization faces the disadvantage of difficult separation from the product [7]. The immobilized cells are easy to fall off from the carrier in the fermentation process, which has become a restriction factor for the extensive application of CF in this field. Therefore, a variety of methods were adopted to overcome the defects of CF by modification or compound methods so as to enhance the binding force between the CF and cells. It has become a trend in the immobilization field to develop composite carrier materials with excellent performances by combining functions, complementing, and optimizing the properties of two or more materials. In our research, the surface of CF was modified using the rapid microwave-assisted phosphoric acid treatment [8]. The CF, which is rich in hydrophilic groups and increases the content of acidic groups after being treated with nitric acid, was used as the cells immobilization carrier for xylitol fermentation [7]. The activity functional groups with the C–P–O and C–O–P bonds on the CF surface increased [7,8]. The improved hydrophilicity and specific surface area enhanced the biocompatibility, resulting in the immobilization efficiency of CF on the microbial cells significantly improved. In addition, the CF surface was modified by carbon stripping and regrowth of in-situ-grown graphene for cells immobilization with the high xylitol yield [8]. The preparation of calcium alginate beads immobilized with *Pseudomonas mendocina DT4* was optimized to effectively treat the tetrahydrofuran-containing wastewater in a packed bed reactor, achieving a removal efficiency of more than 90% [9]. 

Polyurethane foam (PUF) is a kind of high polymer that is formed by polycondensation of polyols and polyisocyanates with the excellent mechanical properties. The PUF particles, as immobilized carriers, were non-toxic to cells and were difficult to decompose by microorganisms and microbial fermentation mediums. It can provide a stable growth environment for immobilized cells to produce the biodiesel fuel by methanolysis of plant oils [10]. The PUF particles have been used for the enzymes or cells immobilized carriers due to their low cost, inertia, and excellent mechanical properties. Using *Candida antarctica* lipase B as a biocatalyst immobilized in polyurethane, the conversion rate of acetic acid in the esterification reaction was improved [11]. The PUF immobilization could serve as an efficient method for improving cell vitality and enzyme reactivity in the continuous operation of fermentation [12]. However, the PUFs also had some disadvantages as carriers, owing to the fact that the surface of PUF is smooth, the pore size is large, the particle adsorption capacity is not high, and the immobilized cells easily fell off [13].

The dopamine (DA, 3, 4-dihydroxyphenylethylamine) is a famous molecular biomimetic adhesive that can interact with almost all types of surfaces due to its self-polymerization, high biocompatibility and hydrophilicity [14]. The DA can self-polymerize in an alkaline solution to form polydopamine (PDA) [15]. The PDA is a biocompatible biomimetic polymer that can form a stable coating on almost any material, hydrophilizing its surface, and is the basis for covalently linking many compounds through thiol or amine groups in a simple water-based process [16,17,18]. In recent years, the covalent or non-covalent interactions between the PDA and biomolecules have been explored. The PDA can become a material in the form of nanoparticles, membranes, and other assemblies in its own right, not just as a coating [19]. The PDA coatings have been repeatedly shown to promote the cell adhesion, proliferation, and biocompatibility. It was shown that compared with the uncoated polycaprolactone nanofibers, the PDA-coated materials promoted the attachment and diffusion of human umbilical vein endothelial cells after incubating cells for 0.5 h [20]. The PDA mainly worked through the covalent bond between the quinone group of PDA (oxidation of catechin groups in dopamine) and the amino group on the surface of microbial cells. Moreover, the microbial cells are fixed on the surface of the immobilized carrier by a Schiff base reaction and Michael-type addition reaction [21]. It was found that the PDA-modified PLA and cadmium selenide quantum dots reduced the inflammatory response of the material to tissues and the immune response of blood [22]. That was, the PDA reduced the toxicity of material to cells in vivo and improved its biocompatibility. It has been shown that the PDA-modified light-induced tunable shape memory of poly (ε-caprolactone)-based PUF had excellent cellular compatibility and wettability [23]. In addition, the appropriate thickness of coating significantly promoted cell adhesion and proliferation. The common immobilization styles of PUF carrier were adsorption, covalent binding, entrapment, affinity immobilization, etc. [24,25], which could apply to the food industry to fabricate various products and goods, including the production of flavors, syrups, confectionaries, milk foods, alcoholic and fruit beverage, yeasts for baked goods, whey lactose hydrolysates [26].

*Candida tropicalis* (*C. tropicalis*) is an ideal microorganism that can use xylose as the sole carbon source and metabolize xylose quickly. It grows rapidly with the high biomass concentration and can carry out growth and metabolic fermentation. It was an ideal strain that can naturally ferment xylose to produce xylitol [27]. Although the bioconversion of xylitol had broad development and application prospects, there are some problems that need to be solved in the process of practical research and industrial promotion, such as how to prepare the novel composite carrier for enhanced cells immobilization to improve the production of xylitol with the relative mechanism on cells immobilization for xylitol fermentation by the novel carrier [3]. After the CF of cells immobilization carriers modified by the rapid microwave-assisted phosphoric acid treatment [7] and nitric acid treatment [8], both the immobilized efficiency and xylitol fermentation were enhanced.

In this paper, the CF powder filled the holes in PUF by ultrasonic dispersion. Then, a layer of PDA film was coated on the surface of the composite materials, which not only improved the hydrophilicity but also strengthened the composite of the CF powder and PUF, avoiding the shortcoming of CF powder falling off easily in the fermentation process as the immobilized carrier. The surface of the composite was modified by the PDA with a large number of amino and carboxyl groups. The PDA endowed the composite carrier with higher biocompatibility, which effectively reduced the reactive oxygen species content of immobilized cells and improved the mitochondrial membrane potential. The composite carrier material was beneficial to the complementary material properties, good biocompatibility, high stability, and mechanical strength. The cell immobilization efficiency of the PDA-CF/PUF composite carrier was significantly improved, resulting in the elevated multi-batch fermentation of xylitol for the application of the important food sweetener and multifunctional food additive.

## 2. Materials and Methods

### 2.1. Materials, Strains and Medium

The *C. tropicalis* used in this experiment was from the laboratory preservation. The PUF (Beijing Hengyuexing Foam Factory, Beijing, China) was low-density, flexible, and open-celled foam, with an average density of 0.02 g (cm^3^)^−1^, a porosity of 90–95%, an average pore size of 0.04 mm, and a side cubic length of 0.4–0.5 cm pieces.

Liquid seed medium: xylose (Analytical Reagent, Fukuda Biotechnology Co., Ltd., Qingdao, China), 20 g·L^−1^; glucose (Analytical Reagent, Tianjin Komio Chemical Reagent Co., Ltd., Tianjin, China), 2 g·L^−1^; yeast extract powder (Analytical Reagent, Beijing Oberstar Biotechnology Co., Ltd., Beijing, China), 10 g·L^−1^; KH_2_PO_4_ (Analytical Reagent, Tianjin Komio Chemical Reagent Co., Ltd., Tianjin, China), 15 g·L^−1^; (NH_4_)_2_SO_4_ (Analytical Reagent, Tianjin Komio Chemical Reagent Co., Ltd., Tianjin, China), 3 g·L^−1^; MgSO_4_·7H_2_O (Analytical Reagent, Tianjin Komio Chemical Reagent Co., Ltd., Tianjin, China), 1 g·L^−1^. Natural pH. Fermentation medium: xylose, 90 g·L^−1^; glucose, 10 g·L^−1^; yeast extract powder, 20 g·L^−1^; KH_2_PO_4_, 15 g·L^−1^; (NH_4_)_2_SO_4_, 3 g·L^−1^; MgSO_4_·7H_2_O, 1 g·L^−1^. Natural pH.

### 2.2. Preparation of Carrier

The CF powder (Shanghai Biyuntian Biotechnology Co., Ltd., Shanghai, China) and PUF were pretreated in acetone solution for 5 h and then washed with 95% ethanol solution (Xinxiang Sanwei Disinfection Preparations Co., Ltd., Xinxiang, China) and deionized water several times. Then, the CF powder and PUF were dried to reserve.

**Figure 1:**
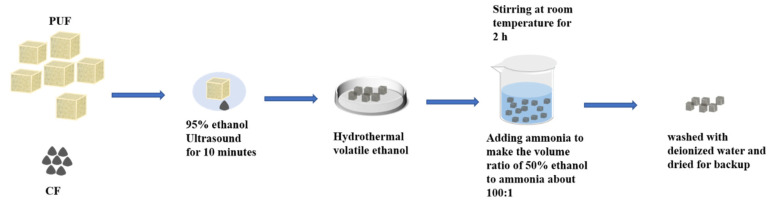
The schematic diagram of the composite carrier preparation process.

The Composite and modification of CF powder and PUF: 1 g of CF powder and 1 g of PUF were ultrasonically mixed in 100 mL of 95% ethanol solution for 10 min, then removed and heated in a water bath at 70 °C to volatilize the ethanol. The carrier was placed in 100 mL 50% ethanol solution with adding ammonia (Tianjin Komio Chemical Reagent Co., Ltd., Tianjin, China) at the volume ratio of 50% ethanol to ammonia about 100:1. A certain mass of the DA hydrochloride (Shanghai Aladdin Biochemical Technology Co., Ltd., Shanghai, China) was added to make the concentration 2 mg·mL^−1^, stirring at room temperature for 2 h. After the reaction, the carrier was collected by filtering out liquid, heated in a water bath for preliminary drying at 70 °C, then washed with the deionized water and dried at 50 °C overnight (the drying loss was less than 3%) to reserve. The schematic diagram of the composite carrier preparation process is shown in Figure 1.

The reaction product with only CF powder added was denoted as CF. The reaction product with only PUF added was denoted as PUF. The reaction product with the CF powder and PUF added was denoted as CF/PUF. The reaction product with the DA hydrochloride, CF powder, and PUF added was denoted as PDA-CF/PUF.

### 2.3. Structural and Performance Characterization of Composite Carriers

The surface morphology of the carrier and the immobilized cell morphology were detected by scanning electron microscope (SEM, Quanta 250FEG, FEI, Hillsboro, OR, USA). The surface functional groups of the carrier were detected by Fourier transform infrared spectroscopy (FTIR, WQF-530, Beijing Beifen Ruili Analytical Instrument Factory, Beijing, China). The surface elemental composition and chemical state of the carrier were detected by X-ray photoelectron spectroscopy (XPS, Nexsa, ThermoFisher, Waltham, MA, USA). The chemical structure of the carrier was detected by an X-ray diffractometer (XRD, Miniflex 600, Nippon Science Company, Tokoy, Japan). The hydrophilicity of the carrier surface was tested by a contact angle tester (YIKE-360A, Chengde Yike test Instrument Factory, Chengde, China).

### 2.4. Biocompatibility Analysis of Composite Carrier

#### 2.4.1. Hydrophilicity Detection of Carrier

The hydrophilicity level of the composites prepared in this experiment was mainly analyzed by the horizontal contact angle of the material. The droplet method for measuring horizontal contact angle steps: the appropriate size of the sample was fixed on the contact angle measuring instrument glass stage, room temperature 25 °C. The deionized water was evenly dropped on the surface of the sample, and the water droplets were measured after dropping. The static water contact angle measuring instrument was used to observe the shape of the droplet. The digital camera was used to take the images. The images were processed, and the contact angles of the droplet were calculated.

#### 2.4.2. Cell Viability by MTT Assay

The immobilized *C. tropicalis* cells of the logarithmic phase on the surface of carriers were washed with 0.1 M, pH 7.4 PBS buffer solution and centrifuged at 1789× *g* for 5 min at 4 °C to collect the precipitation. The fresh PBS buffer solution was added. The cells were diluted to about 5–10 × 10^4^ cells·mL^−1^ by cells counting. According to the previous method [28], the effect of the carrier on the viability of immobilized cells was determined by MTT (3-(4,5-dimethyl-2-thiazolyl)-2,5-diphenyl-2-H-tetrazolium bromide).

#### 2.4.3. Cell Viability by Confocal Laser Scanning Microscope (CLSM)

Using Acridine Orange (AO) and Propidium Iodide (PI) as the staining solution, CLSM was used to detect the effect of different carriers on immobilized cell viability [29]. The specific steps were as follows: After 24 h of cells fermentation, the immobilized cells on the surface of the carrier were washed with 0.1 M, pH 7.4 PBS buffer solution. The immobilized cells were collected by the centrifugation at 1789× *g* for 5 min at 4 °C. The residual medium was removed by the centrifugation and washed with the PBS buffer solution. The cell concentration was adjusted to 5 × 10^6^ cells ·mL^−1^ by cells counting. A volume of 1 mL of the above concentration of cells fluid was taken and centrifuged. The cells were precipitated. The supernatant was carefully aspirated, and 0.5 mL of mixed staining solution (containing AO 0.05 mg·mL^−1^, PI 0.05 mg·mL^−1^) was added. The cells were incubated at room temperature without light for 10 min. After removing the unbound dyeing solution by the centrifugation and washing, 1 mL 4% paraformaldehyde fixative was added and fixed at 4 °C for 15 min. The paraformaldehyde was removed by the centrifugation, and the cells were suspended by adding 1 mL PBS buffer.

#### 2.4.4. ROS Levels by Flow Cytometry

According to the method described in reference [30], the reactive oxygen species (ROS) levers were detected as follows. The treatment of cells before staining is described in Section 2.4.2. Then, 0.2 mL of 20 μmol·L^−1^ DHR123 was added. The mixture was gently blown and mixed to incubate at 37 °C for 1 h away from light. After centrifugation and washing with the PBS buffer for one time, 0.5 mL PBS was added to resuspended cells. Then, the ROS content was detected by the flow cytometry.

#### 2.4.5. Mitochondrial Membrane Potential by Flow Cytometry

According to the method described in the literature [31], the mitochondrial membrane potential was detected as follows. The treatment of cells before staining is described in Section 2.4.2. Then, 0.2 mL JC-1 (Shanghai Biyuntian Biotechnology Co., Ltd., Shanghai, China, Analytical Reagent, ≥98%) staining solution was added to incubate at 37 °C for 20 min under dark conditions. After the centrifugation and washing with PBS buffer for one time, 0.5 mL PBS was added to resuspended cells. Then, the mitochondrial membrane potential was detected by the flow cytometry.

### 2.5. Effects of Composite Carrier on Biomass and Cell Immobilization Efficiency

*C. tropicalis* were immobilized on a composite carrier. *C. tropicalis* was extracted from 4 °C plate medium and incubated in seed medium to logarithmic phase (18 h at 30 °C, 160 r·min^−1^). Then, 10% (*v*·*v*^−1^) of the inoculum in the pre-culture was transferred to carrier-free and fermentation medium bottles containing CF, CF/PUF, and PDA-CF/PUF carriers. *C. tropicalis* cells were immobilized on the carriers of PUF, CF/PUF, and PDA-CF/PUF by the natural adsorption, respectively, which were incubated under the same culture conditions.

The biomass included immobilized cell biomass and free cell biomass. After the fermentation, the fermentation broth was filtered out to obtain the immobilized cells, which were washed with the deionized water to remove the free cells and dried to constant weight with the drying oven at 50 °C. Then, the increase in mass of the carrier was taken as the biomass of the immobilized cells. The remaining fermentation liquid and the above washing liquid were centrifuged together at 4260× *g* for 5 min. The precipitation mass after drying was the biomass of free cells. 

The immobilization efficiency of the carrier to the cells was the dry weight of the cells fixed by the unit mass carrier. The immobilized carrier with a certain mass (*m*_0_) was added to the fermentation culture to immobilize cells. After the fermentation, the carrier and immobilized cells were taken out, and the free cells on the surface were washed with deionized water. After drying to a constant weight, the total mass of the carrier and immobilized cells (*m*_1_) was weighed. Then, the immobilization efficiency was calculated as IE =$\frac{m_{1}-m_{0}}{m_{0}}$.

### 2.6. Effects of Composite Carrier on Multi-Batch Fermentation of Immobilized Cells

*C. tropicalis* cultured on a 4 °C plate was placed in a 250 mL shake flask containing 50 mL seed medium to prepare a seed medium pre-culture. After the incubation at 30 °C and 160 r·min^−1^ for 18 h, 10% (*v·v*^−1^) inoculum of the pre-culture was added to a 250 mL shake flask containing 50 mL fermentation medium and divided into PUF, CF/PUF, and PDA-CF/PUF carriers. *C. tropicalis* cells were immobilized on the carriers of PUF, CF/PUF, and PDA-CF/PUF by the natural adsorption.

The xylitol fermentation experiments of *C. tropicalis* were carried out in batches of 48 h. At the end of each batch, the fermentation broth was collected and replaced. The PUF, CF/PUF, and PDA-CF/PUF carriers with immobilized cells were left for the next batch. Then, new media was added. The fermentation broth was centrifuged at 4260× *g* for 10 min. The supernatant was collected, and the content of xylitol was detected. Then, the yield of xylitol was calculated. The content of xylitol was detected by HPLC instruments (Shimadzu LC-2030C liquid chromatograph, Kyoto, Japan). The specific detection scheme is as follows:

The peak time and peak area of the xylitol standard solution were detected by HPLC with a differential refractive index detector (RID-20A). The chromatographic column was a Sugar column (6.5 × 309 mm Waters, Milford, MA, USA), with the mobile phase (purified water) having a flow velocity of 0.5 mL min^−1^ at a column temperature of 80 °C. The sample volume was 10 μL.

According to the peak time of the xylitol standard solution, the peak area changes in all samples were detected at this time. The xylitol yield of all samples was calculated according to the xylitol standard curve. The standard curve was determined: y = 167462x + 1753, R^2^ = 0.9989. The xylitol yield (%) is expressed as the ratio between the final xylitol concentration and the initial xylose concentration in the broth.

### 2.7. Effects of Immobilization on Expression Levels of Genes Related to Xylitol Synthesis

After the culture of 24 h, immobilized cells on different carriers were collected. The xylose reductase, encoded by *XYL1*, was a key enzyme that affected *C. tropicalis* to convert xylose to xylitol [26], catalyzing the reduction of D-xylose to xylitol. According to the NCBI gene sequence, the Primer Premier 5.0 software was used to design the primers. The *ACT1* gene was used as the endogenous reference gene. RNA was extracted using the method previously reported [27]. 1 μg of qualified total RNA was reversely transcribed into cDNA using the kit (PrimeScript™ RT reagent Kit with gDNA Eraser, TaKaRa, Kusatsu City, Japan). The real-time quantitative polymerase chain reaction (RT-qPCR) was performed using the TB Green Premix Ex Taq II (Tli RNaseH Plus, Kusatsu City, Japan) kit (TaKaRa Biotech, Kusatsu City, Japan). The RT-PCR reaction was carried out on an RT-PCR thermal cycler (Tm = 55 °C). The fold changes of target genes were calculated by the 2^−ΔΔCT^ method. Table S1 Primers used in experiments.

### 2.8. Statistical Analysis

The results were statistically analyzed using SPSS 17.0 (SPSS, Chicago, IL, USA). All experiments were carried out in triplicate. Data were analyzed using the ANOVA method, and means were significantly different at *p* < 0.05.

## 3. Results and Discussion

### 3.1. Effect of Surface Morphology Detection of Composite Carrier on Cells Immobilization

**Figure 2:**
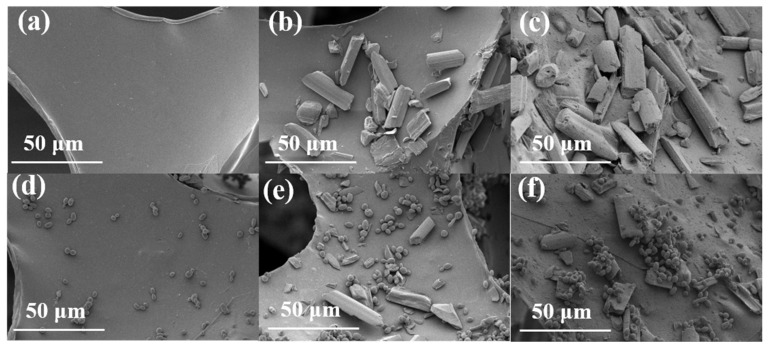
The electron microscopy (SEM) images of PUF, CF/PUF, and PDA-CF/PUF carriers before and after immobilization. (**a**) PUF, (**b**) CF/PUF, (**c**) PDA-CF/PUF, (**d**) PUF immobilized cells, (**e**) CF/PUF immobilized cells, (**f**) PDA-CF/PUF immobilized cells.

The surface morphology images of PUF, CF/PUF, PDA-CF/PUF, and immobilized cells were observed by SEM. As shown in Figure 2a, the PUF has a smooth, clean surface with many channels and pore structures. Figure 2b shows that the surface of PUF was loaded with short rods of the CF powder, which modified the pore structure of the polyurethane. This might help to avoid the risk of cell shedding caused by smooth polyurethane surfaces during fermentation. Figure 2c shows that compared with the CF/PUF, PDA-CF/PUF was loaded with a membrane structure, indicating that the PDA coating provided a protective film for the CF/PU composite carrier. Figure 2d showed that the untreated PUF loaded only a small amount of yeast cells on the smooth surface. Figure 2e showed that the rough surface of CF/PUF was adhered with more yeast cells. Figure 2f showed that the PDA loaded a layer of membrane structure on the surface, and the PDA-CF/PUF immobilized most yeast cells. The immobilized cells on the PDA-CF/PUF are all on the surface of the internal pore structure, which has a strong affinity for the immobilized cells, providing the high activity for cell growth and xylitol fermentation. It was shown that the PDA formed a covalent or non-covalent bond (hydrogen bond or van der Waals force) with the surface of the matrix material through the catechol functional group [23], thus forming a stable PDA-CF/PUF composite carrier. In addition, it could be seen from the SEM images that there was no shrinkage in the foam during the preparation of the composite carrier PDA-CF/PUF.

Different numbers of *C. tropicalis* cells were fixed on the surfaces of PUF, CF/PUF, and PDA-CF/PUF. Among them, more yeast cells adhered to the surface of PDA-CF/PUF, which was due to the fact that the PDA could enhance the adhesion of materials to cells [23]. The PDA could enhance the focal adhesion of cells and promote cells proliferation with high biocompatibility. The active catechol and amine groups in the PDA allowed yeast cells to further use biomolecules (such as avidin and exogenous flavin mononucleotides) for functionalization [19]. The PDA also provided rich chemical properties, allowing the post-functionalization of the obtained coating with metal nanoparticles. Through the self-polymerization of DA, the PDA coating with strong adhesion was formed on a variety of materials. PDA functions as a material-independent, multifunctional surface modification reagent that could also serve as polydopamine–melanin initiators for surface-initiated ATRP (atom transfer radical polymerization) [32,33,34]. Facile conjugation of biomolecules onto surfaces was achieved through the use of mussel adhesive protein-inspired coatings, facilitating efficient protein adsorption onto dopamine–melanin films. This approach offered a robust and biocompatible method for surface modification and immobilization of biomolecules [35,36].

The results showed that the smooth surface of PUF was modified by filling the CF. The presence of PDA enhanced the loading of CF on the PUF surface. Cells were on the surface of the composite carrier. Thus, the PDA-CF/PUF composite carrier could achieve higher cell immobilization efficiency.

### 3.2. Influence of Hydrophilicity and Chemical Structure of Composite Carrier on Cells Immobilization

**Figure 3:**
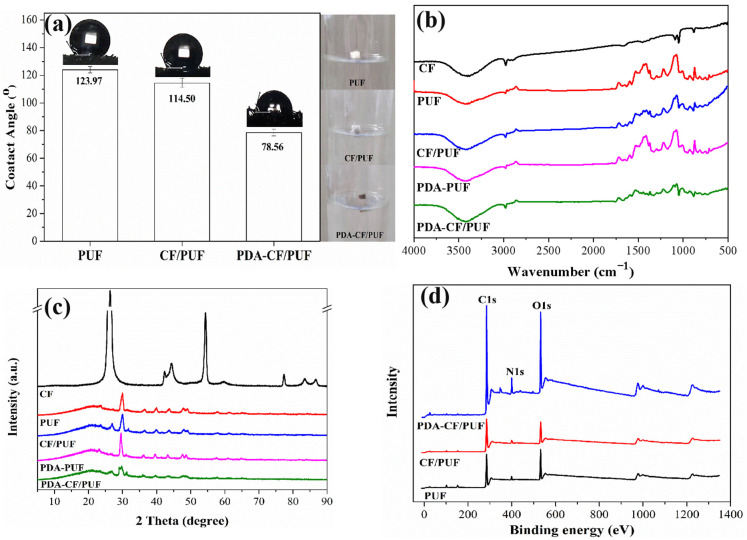
(**a**) The contact angle analyses of PUF, CF/PUF, and PDA-CF/PUF carriers. (**b**) The Fourier transform infrared spectroscopy (FTIR) spectrums of CF, PUF, CF/PUF, and PDA-PUF carriers. (**c**) The X-ray diffraction (XRD) patterns of CF, PUF, CF/PUF, PDA-PUF, and PDA-CF/PUF carriers. (**d**) The X-ray photoelectron spectroscopy (XPS) spectrums of CF/PUF and PDA-CF/PUF carriers.

As shown in Figure 3a, the PUF was a material with strong hydrophobicity, with a contact angle of 123.97°. The hydrophilicity increased slightly after loading the CF, and the contact angle decreased by 7.64%. However, the modification of PDA further increased the hydrophilicity of the CF/PUF composite carrier, resulting in the contact angle decreasing by 36.63%, compared with the PUF. It may be due to the fact that the PDA, as a widely used biocompatible surface modification reagent, could introduce hydrophilic hydroxyl and amino groups onto the surface of the material [23], which improved the hydrophilicity and biocompatibility [37]. The PDA increased the wettability of the material and promoted the rate of adhesion and spread of fibroblasts [18]. The moderate hydrophilicity on the surface of the immobilized carrier was conducive to the immobilization of *C. tropicalis* cells [8]. In addition, the improvement in hydrophilicity was beneficial to the physiological activity of immobilized cells [38]. In conclusion, the PDA-CF/PUF was a novel composite carrier with broad application potential in the field of cells immobilization for enhanced cells immobilization.

Figure 3b shows the FTIR images of each carrier in the range of 500–4000 cm^−1^. The absorption peaks of CF at 3423 cm^−1^ and 1662 cm^−1^ were related to the OH tensile vibration [39] and C=O tensile vibration [40], respectively. In the spectra of PUF, CF/PUF, and PDA-CF/PUF, the vibration absorption peaks located at 3300 cm^−1^, 1652 cm^−1^, and 1531 cm^−1^ belonged to the N–H group, CO–NH group, and –NH group, respectively, proving the existence of ammonia ester group [41]. In addition, the absorption peak at 1717 cm^−1^ corresponded to the C=O tensile vibration [42]. The strong peak at 1220 cm^−1^ corresponded to the C–O–C group [43]. The spectra showed that the chemical structure characteristics of PUF were preserved after loading the CF or modification with the PDA. Compared with the PUF, the PDA-PUF and PDA-CF/PUF had stronger absorption peaks at 3200–3700 cm^−1^, which was due to an increase in N–H groups and O–H groups after covering the PDA film. It reported that active groups on the surface of PDA, such as catechol, amine, imine, and hydroxyl, could interact with polyurethane chains to form strong hydrogen bonds [23,44]. In addition, the characteristic peak strength of CF/PUF and PDA-CF/PUF polyurethane was relatively weakened due to the loading of the CF. The results showed that the PDA coating had been successfully loaded on the surface of the CF/PUF composite carrier, which improved the cell affinity to enhance cells proliferation and immobilization on the material [23]. The composite carrier material was beneficial to the complementary material properties with good biocompatibility, high stability, and mechanical strength.

Figure 3c shows the XRD patterns of the five carriers (CF, PUF, CF/PUF, PDA-PUF, and PDA-CF/PUF) at 2θ = 5~90°. The diffraction peak of CF appeared in the range of 20~30° [45]. The wide peak at 26° could be attributed to reflective surface C (002) with disordered aromatic carbon structure, while the characteristic peak at 43.4° came from reflective surface C (100/101) of graphite or organic carbon [46].

For the PUF, there was a wide diffraction peak in the range of 15~25°, with the maximum peak at 21° [47]. For the CF/PUF and PDA-CF/PUF, when the CF was loaded on the surface of PUF, the two characteristic diffraction peaks of CF overlapped with the peaks of PUF, with their decreased intensity. This was because the addition of CF destroyed the original uniform structure of PUF and made the CF/PUF more amorphous, leading to a reduction in strength [47].

In addition, the PDA-PUF and PDA-CF/PUF did not observe characteristic peaks of PDA, which might be due to the poor crystallinity and the thin coating of PDA [48]. It was suggested that the PDA layer on the carrier surface had little effect on the XRD patterns [49]. Therefore, in order to further explore the effect of polydopamine on the chemical structure of the carrier, the XPS analysis was carried out on the carrier.

The surface element compositions of the carriers were determined by XPS. The XPS images of PUF, CF/PUF, and PDA-CF/PUF are shown in Figure 3d. The surface element compositions of the carriers are shown in Table S2. The full scan spectrum of the carriers included C1s, N1s, and O1s peaks. Previous studies have shown that the N/C ratio of PDA was about 12% [50,51]. As shown in Table S2, the N/C ratio of PDA-CF/PUF (7.91%) was significantly higher than that of the CF/PUF (5.83%). The results showed that the PDA has been successfully loaded on the surface of the CF/PUF carrier to the PDA-CF/PUF.

### 3.3. Effect of PDA-CF/PUF Composite Carrier on Biocompatibility of Immobilized Cells and Expression of Genes Related to Xylitol Production

**Figure 4:**
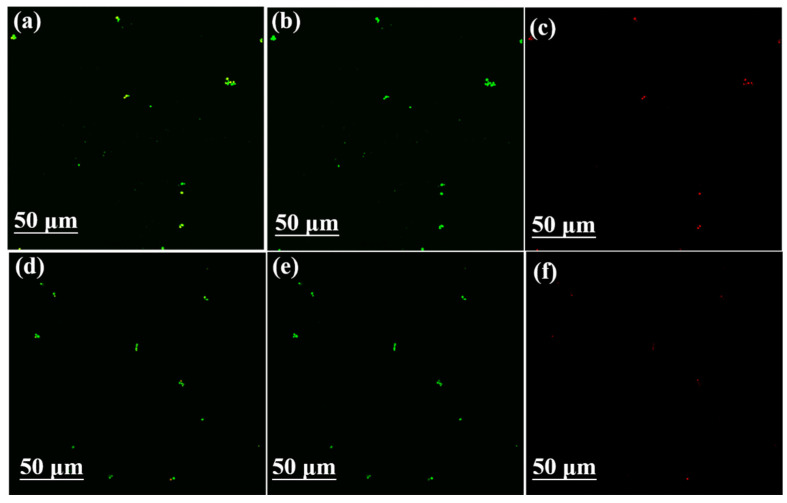
CLSM images of different carriers of immobilized *C. tropicalis* by PI and AO. (**a**) The immobilized cells on the carrier of CF/PUF (showing green and red fluorescence), (**b**) The immobilized cells on the carrier of CF/PUF (showing green fluorescence only), (**c**) The immobilized cells on the carrier of CF/PUF (showing red fluorescence only), (**d**) The immobilized cells on the carrier of PDA-CF/PUF (showing green and red fluorescence), (**e**) The immobilized cells on the carrier of PDA-CF/PUF (green fluorescence only), (**f**) The immobilized cells on the carrier of PDA-CF/PUF (red fluorescence only).

In order to determine the effect of PDA-CF/PUF composite carriers on the viability of immobilized cells, CLSM detections were performed on the CF/PUF and PDA-CF/PUF immobilized cells. As shown in Figure 4, compared with the CF/PUF, PDA-CF/PUF immobilized *C. tropicalis* showed more green fluorescence (live cells) and less red fluorescence (damaged/dead cells) [29], suggesting that the PDA improved the biocompatibility of carrier materials to immobilized cells.

**Figure 5:**
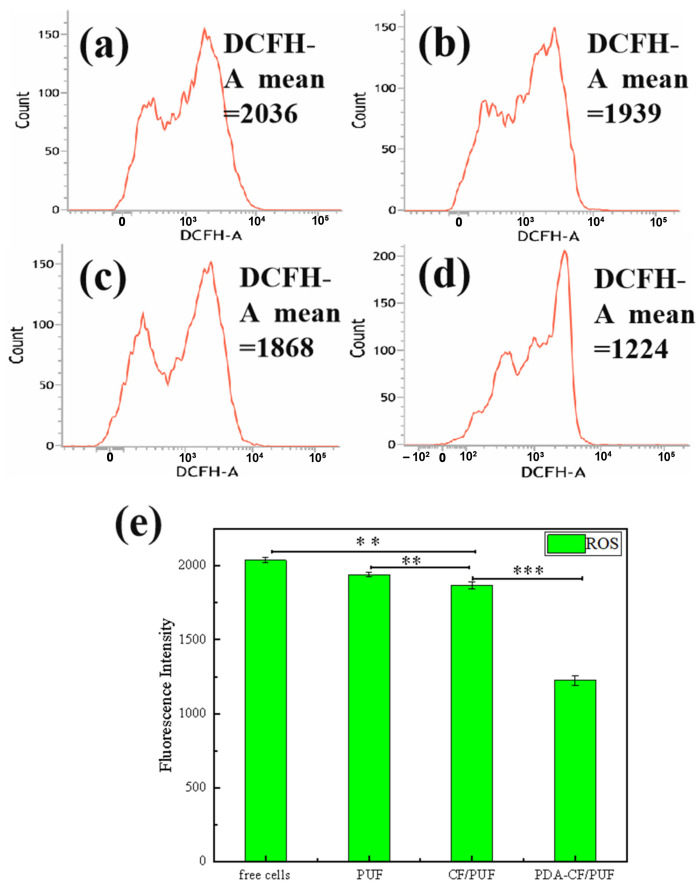
The effects of different carriers on reactive oxygen species (ROS) levels of immobilized cells. (**a**) free cells, (**b**) PUF immobilized cells, (**c**) CF/PUF immobilized cells, (**d**) PDA-CF/PUF immobilized cells, (**e**) fluorescence intensity of free cells, PUF immobilized cells, CF/PUF immobilized cells, and PDA-CF/PUF immobilized cells. The signal of *** means *p* < 0.001. The signal of ** means *p* < 0.01.

Excessive ROS levels can lead to oxidative stress and membrane damage of microbial cells, thereby promoting cell apoptosis [30]. DHR123 can be oxidized by ROS to Rh123 with the fluorescence. The ROS level can be reflected by measuring the average fluorescence intensity of Rh123 in cells by the flow cytometry. As shown in Figure 5, the average fluorescence intensity of Rh123 in the descending order was: free cells > PUF immobilized cells > CF/PUF immobilized cells > PDA-CF/PUF immobilized cells. Therefore, lower ROS levels were detected under the immobilized *C. tropicalis*. Among them, the ROS level was the lowest in the cells immobilized by the PDA-CF/PUF due to the high biocompatibility and ROS clearance ability with the PDA [52]. The enhancement of immobilization capacity was accompanied by an increase in the ability to remove the ROS and an increase in biocompatibility [53].

**Figure 6:**
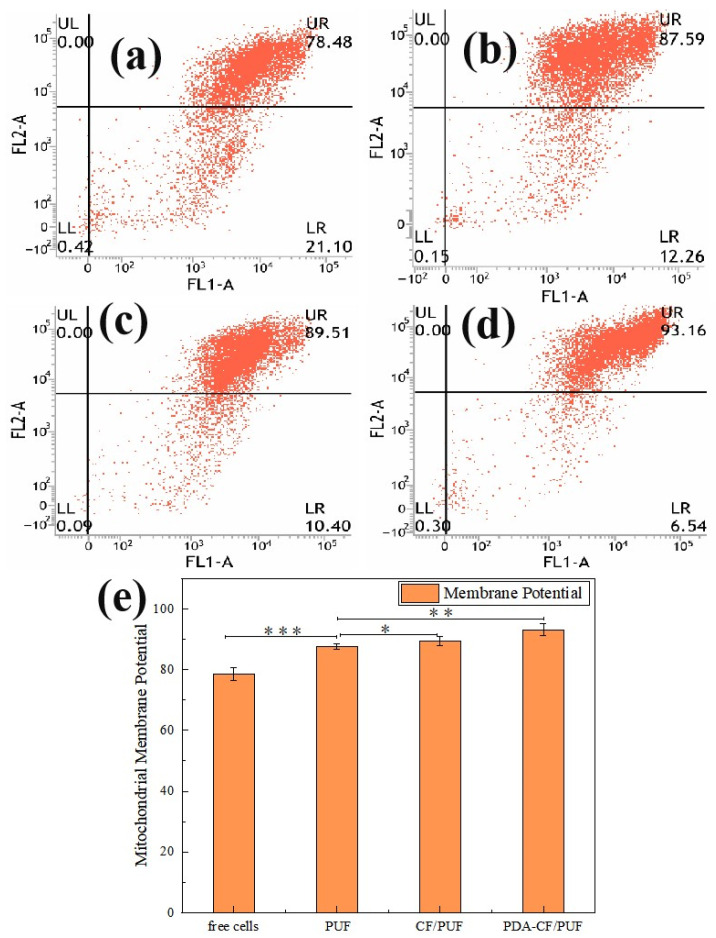
The effects of different carriers on mitochondrial membrane potential of immobilized cells (**a**) free cells, (**b**) PUF immobilized cells, (**c**) CF/PUF immobilized cells, (**d**) PDA-CF/PUF immobilized cells, (**e**) mitochondrial membrane potential of free cells, PUF immobilized cells, CF/PUF immobilized cells and PDA-CF/PUF immobilized cells. The signal of *** means *p* < 0.001. The signal of ** means *p* < 0.01. The signal of * means *p* < 0.05.

As shown in Figure 6, the order of mitochondrial membrane potential from large to small was PDA-CF/PUF immobilized cells > CF/PUF immobilized cells > PUF immobilized cells > free cells. The mitochondrial membrane potential could induce the release of apoptotic proteins to promote cell apoptosis. The protection of mitochondrial membrane potential plays an important role in anti-apoptosis; the higher the mitochondrial membrane potential, the more living cells there are [31]. Therefore, cells immobilized with the PDA-CF/PUF had higher cell activities than those of the CF/PUF immobilized cells, PUF immobilized cells, and free cells. The results of MTT, CLSM, and flow cytometry showed that the PDA-CF/PUF was the immobilized carrier material with the highest biocompatibility and affinity, which was more in line with the requirements of biological materials [54].

It could be seen from Figure S1 that when the initial pH value was 4.0, the ability of yeast to utilize xylose was significantly inhibited. When the initial pH value was 7.0, the xylose consumption ability decreased significantly, and the residual sugar content increased, which was not conducive to the fermentation of the bacteria. Therefore, pH 6.0 is the optimum fermentation pH condition.

**Figure 7:**
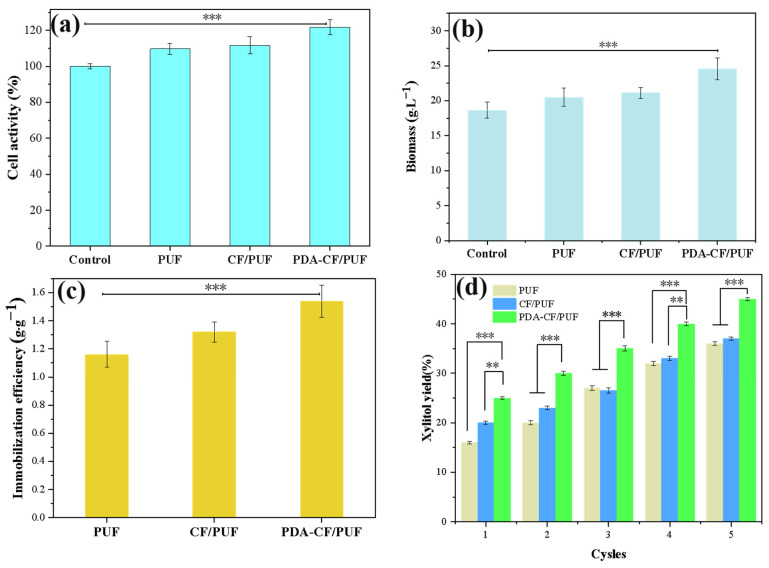
The effects of different carriers on immobilized cells. (**a**) The effects of different carriers on immobilized cell activity. (**b**) The effects of different carriers on biomass. (**c**) The effect of different carriers on cell immobilization efficiency. (**d**) The effects of different carriers immobilized cells on xylitol fermentation. The signal of *** means *p* < 0.001. The signal of ** means *p* < 0.01.

The MTT assay is used to detect the effect of each carrier on the viability of immobilized cells. The MTT assay evaluates cell survival and growth by detecting the succinate dehydrogenase of mitochondria in living cells to convert the exogenous MTT into water-insoluble blue-violet crystal formazan [55]. Dead cells cannot complete this transformation. As shown in Figure 7a, the cell activities of PUF immobilized cells, CF/PUF, and PDA-CF/PUF increased gradually, which were higher than those of the free cells. It might be due to the fact that the immobilization by the carriers could provide protective sites for cells growth and proliferation [54]. The relative viability of PDA-CF/PUF cells was the highest, probably because the PDA had a significant positive effect on improving cell viability. As the cell immobilized carrier, the composite material could be prepared with a larger controllable size, which was similar to the previous literature [22,56].

As shown in Figure 7b, the cell biomass of free cells, PUF, CF/PUF, and PD-CF/PUF were 17.58, 19.28, 19.90, and 23.15 g·L^−1^, respectively. Compared with the free cells, the cell biomass with the immobilized carriers of PUF, CF/PUF, and PD-CF/PUF was increased by 9.97%, 13.19%, and 31.69%, respectively, which might be due to the protection of immobilized cells provided by carriers and the adsorption of nutrients on the surface of carriers. Thus, it could be more easily used by the bacteria for growth and proliferation [16]. Therefore, the modification of PDA improved the biocompatibility of the carrier. By reducing the intracellular ROS level to ensure the improvement of immobilized cell viability and biomass, the fermentation efficiency could be effectively improved [57].

As shown in Figure 7c, compared with the PUF, the cell immobilization efficiency of CF/PUF increased slightly (13.70%). The PDA-CF/PUF significantly increased the cell immobilization efficiency (32.56%), which was related to an increase in hydrophilicity and the content of active functional groups induced by PDA. Therefore, the PDA-CF/PUF carrier could be used as an immobilized material with a high affinity for cells. The biocompatibility of the carrier was improved, with the enhanced immobilized efficiency and fermentation [8].

As shown in Figure 7d, the high xylitol yield was obtained by the immobilized cells on each carrier for the multi-batch fermentation. In general, the xylitol yield of PDA-CF/PUF immobilized cells was significantly higher than that of the other two carriers, because the higher biocompatibility was conducive to cells growth and viability [30]. In the first two batches, the xylitol yields produced by immobilized cells on the CF/PUF and PDA-CF/PUF were both higher than that of PUF. In the third to fifth batches, the xylitol yields produced by immobilized cells on the CF/PUF and PUF carriers were close. The xylitol yields on the PDA-CF/PUF were the highest. During the seven batches of xylitol fermentation, the xylitol yields by the immobilized cells on the carriers of PDA-CF/PUF were higher than those of CF/PUF and PUF. The xylitol yield by the immobilized cells on the PDA-CF/PUF reached the maximum in the fifth batch, with the xylitol yield of 45.32%. The average yield of xylitol fermentation by the PDA-CF/PUF during the seven batches was 37.53%, which was higher than either CF/PUF or PUF. The carrier provides a good growth environment for immobilized cells, with a high affinity for cells, which benefits cells growth and proliferation. 

Additionally, the carrier could play a protective role for the immobilized cells, which is conducive to the stable immobilization of cells for fermentation. Improving the biocompatibility of the carrier can give the immobilized cells higher fermentation efficiency and increase fermentation production [33,57]. The previous literature reported the ultrasonic enhancement of xylitol production from sugarcane bagasse using *C. tropicalis* MTCC 184 immobilized on PU foam. The xylitol yield of 0.66 g/g of xylose has been obtained in the ultrasound-assisted fermentation in just 15 h [58]. Another literature study reported that the immobilized *C. tropicalis* cells in freeze-dried calcium alginate beads were used for the production of xylitol. In five batches, *C. tropicalis* produced 34 g/L xylitol with a yield of 0.67 g/g [59]. Thus, the immobilized cell fermentation is an effective method to improve the production of xylitol. It is worth noting that the xylitol yield of immobilized cells increased first and then decreased during seven batches of fermentation. In the first five batches of immobilized fermentation, the xylitol yield gradually increased until the maximum, indicating that the immobilized cells showed the appropriate and continuous proliferation, with the high enzyme activity to accumulate the xylitol [12]. After the 6th batch of immobilized fermentation, with the performance of more batches, the risk of leaking immobilized cells increased. The autolysis and plasmolysis of cells began to intensify, resulting in the increased liquid viscosity of the culture, with a reduction in cell viability and xylitol production [12,28]. In conclusion, the PDA-CF/PUF was the immobilized carrier with the high biocompatibility and adhesion properties, which had great development potential in the industrial production of xylitol through batch fermentation.

**Figure 8:**
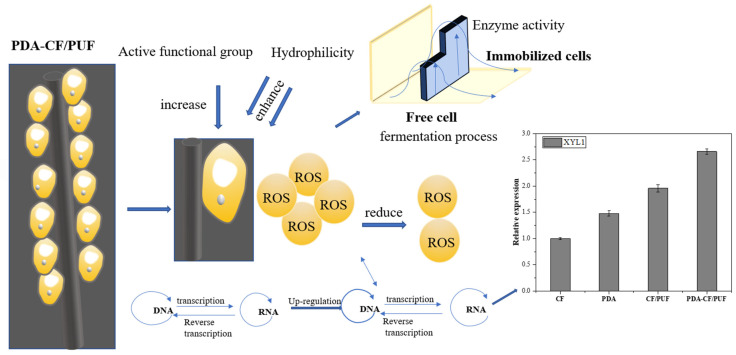
Immobilization mechanism of the composite carrier of PDA-CF/PUF to improve cells immobilization and xylose fermentation.

Immobilized carriers may affect genes related to microbial fermentation product synthesis [33] and thus increase the yield of xylitol. Therefore, the real-time quantitative PCR was used to compare the effects of different carriers on the expression levels of genes related to xylitol synthesis in immobilized cells. As shown in Figure 8, the expression of the *h* gene encoding xylitol was 2.5 times that of free cells, respectively. Under the immobilization condition of the PDA-CF/PUF solid composite, the number of surface-active functional groups increased, which was caused by an increase in N–H and O–H groups after covering the polyamine film [23]. Others reported that the surface-active groups of polyamines, such as catechol, amine, imine, and carboxyl, could interact with the polyurethane chain to form strong hydrogen bonds [57]. 

The load of the PDA layer and the CF/PUF on the carrier surface enhanced the value increment and immobilization of cells on the material and endowed the material with good cell affinity [23]. Among them, the ROS level in PDA-CF/PUF fixed cells was the lowest because PDA had good biocompatibility and the ability to remove ROS [30]. The upregulation of *XYL1* expression may be related to the removal of reactive oxygen species under immobilization. It was reported that the performance of organisms could be improved by improving the biological regulation ability and reducing the accumulation of ROS [60]. The immobilization of composite material PDA-CF/PUF increased the xylitol yield, which was realized with the upregulation of *XYL1* gene expression. This showed the importance of the effect of immobilized carriers on the yield of yeast cells. It was suggested that immobilization might increase the expression of related enzymes such as *acyB* in Streptomyces temperature-resistant [61].

It was found that the change in SOD activity was caused by growth under a fixed state [62]. The embedded cells showed that the activity of superoxide dismutase (SOD) increased more than 1.5 times, and the activity of catalase increased moderately. The observed increase in enzyme activity was related to the de novo synthesis of proteins in fungal cells, which was a necessary condition for the immobilization reaction [47]. During the immobilized fermentation of *Clostridium acetobutylicum*, most of the genes (*hydGEF*, *hydA1*, *hydA2*, *fhuBDC*, and *CA_P0141-CA_P0142*) involved in encoding hydrogenase, NADH-ferrioxidoreductase, and ferrioxidoreductin were upregulated in gene expression intensity. The results showed that the use of composite PDA-CF/PUF to immobilize cells enhanced gene expression. Specifically, the upregulation of the XYL1 gene played a crucial role in improving xylitol transformation. In addition, the composite carrier of PDA-CF/PUF had the highest immobilization efficiency for *C. tropicalis* with high biocompatibility. The innovative composite carrier could improve enzyme activity with cells immobilization, leading to an increase in gene expression levels and production yields through positive feedback loops [31].

The PDA layer was loaded onto the surface of CF/PUF to form the composite carrier of PDA-CF/PUF. The surface-active functional groups of PDA-CF/PUF composites increased. The biomass of immobilized cells was higher than that of free cells, which reflected that the composite carrier had a higher affinity for cells. The fermentation efficiency of immobilized cells was much higher than that of free cells, indicating that the composite carrier improved the cell viability with good biocompatibility. Also, the PDA had a high ability to remove ROS [49,50]. The biomass and fermentation yield of immobilized *C. tropicalis* were higher than those in its free state. The composite carrier reduced the ROS level of *C. tropicalis* and ultimately promoted the upregulation of xylitol synthesis genes. The expressions of *XLl1* of immobilized cells were improved from the carriers of CF, PDA, CF/PUF to PDA-CF/PUF. The prepared novel composite carrier PDA-CF/PUF improved cells immobilization and xylitol fermentation.

## 4. Conclusions

In summary, the PDA-CF/PUF, as a novel composite carrier, was prepared to have high specificity and stability for effective immobilization in xylitol fermentation. The smooth surface and pore structure of PUF were modified by the CF to fill the interior hole of PUF and a layer of PDA film was coated on the surface of the composite carrier. The SEM, contact angle, FTIR, XPS, XRD, MTT, CLSM, and flow cytometry were used to investigate the changes in the structure and properties of the carrier on the effect of the immobilized cells and the immobilization mechanism. The results showed that the composite carrier of PDA-CF/PUF could greatly improve cells immobilization and fermentation efficiency. The surface hydrophilicity of the composite carrier was improved by the abundant active groups of the PDA. The PDA treatment effectively improved the hydrophilicity, active functional groups, and biocompatibility of the composite carrier to improve the cells immobilization and xylitol yield by the PDA-CF/PUF composite carrier. In addition, the PDA-CF/PUF carrier increased the expression level of the *XYL1* gene encoding xylitol in the cells to enhance fermentation. Therefore, the PDA-CF/PUF is a novel composite carrier with the good biocompatibility and immobilization performances. It provides a feasible strategy to improve the fermentation of xylitol and the possibility of large-scale production.

## Data Availability

The original contributions presented in the study are included in the article/Supplementary Materials, further inquiries can be directed to the corresponding authors.

## Supplementary Materials

The following supporting information can be downloaded at: https://www.mdpi.com/article/10.3390/foods13121911/s1, Table S1: Primers used in experiments. Table S2: Surface element composition of different carriers. Figure S1: The effect of different pH on PDA-CF/PUF fermentation. The signal of *** means *p* < 0.001.
