# Supplementary material for: Preparation and Immobilization Mechanism on a Novel Composite Carrier PDA-CF/PUF to Improve Cells Immobilization and Xylitol Production
Source: Foods. 2024 Jun 18;13(12):1911. doi: 10.3390/foods13121911 (PMC11202654; doi:10.3390/foods13121911)
Supplement: Supplementary file 1 [file foods-13-01911-s001.zip › foods-2927547-supplementary.pdf]

Supplementary Materials

Table S1 Primers used in experiments

Gene	Primer	Sequence
ACT1	Forward	5’-CAATACGCCACTGCTGATGC-3’
	Reverse	5’-TGGCGTGAGGTAGAGAGAAACC-3’
XYL1	Forward	5’-GGACTTTCTCTATCCCTTGCGA-3’
	Reverse	5’-TGTCGATCTCTTTATAGTTGTTGGA-3’

Table. S2 Surface element composition of different carriers

Carriers	C	N	O	N/C	O/C
PUF	71.84	4.08	24.08	5.68	33.52
CF/PUF	74.66	4.35	20.99	5.83	28.11
PDA-CF/PUF	71.77	5.68	22.55	7.91	31.42

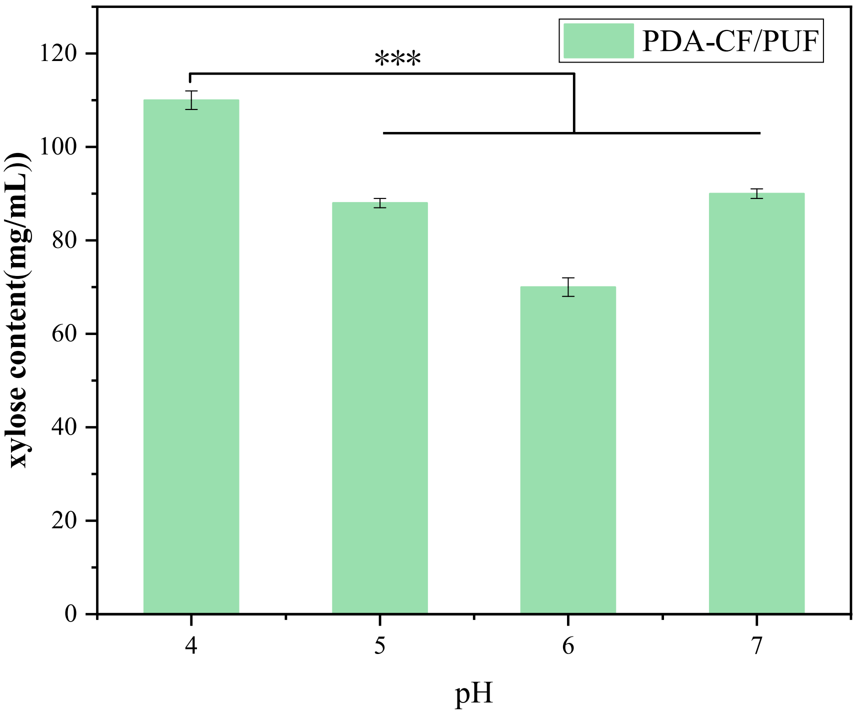

Figure S1. The effect of different pH on PDA-CF / PUF fermentation. The signal of * * * means $p < 0.001$.
